# Calibration and evaluation of Quigley’s hybrid housing price model in Microsoft Excel

**DOI:** 10.1371/journal.pone.0215954

**Published:** 2019-04-25

**Authors:** Alan G. Phipps, Dingding Li

**Affiliations:** 1 Department of Sociology, Anthropology and Criminology, University of Windsor, Windsor, Ontario, Canada; 2 Department of Economics, University of Windsor, Windsor, Ontario, Canada; Xiamen University, CHINA

## Abstract

Quigley derived his hybrid price model to improve the precision of predicted prices of sold homes by statistically merging data of resold homes in a repeat sales model with that of once-sold homes in a single sales hedonic price model. The literature has few applications of the hybrid model aside from those by Quigley and his collaborators. Two reasons for this underuse may be its computational intensiveness and its marginal empirical improvement in comparison with two other models. This paper first demystifies this computational intensiveness by calibrating models in Microsoft Excel with transferable procedures into other software. It second evaluates the hybrid price model’s empirical improvement as a reason for its underuse by predicting prices of 2,559 sold and resold homes observed in two inner-city neighbourhoods in Windsor, Ontario, during a 30-year period. The results as hypothesized are its lower standard errors of regression coefficients and higher simple R-squared than those of a single sales hedonic price model. Moreover, the hybrid model’s predictions have higher correlations than those of the single sales model with not only in-sample observed prices or changes in prices but also out-of-sample ones. The conclusion speculates in plans for future research about reasons for two models’ similar or dissimilar regression coefficients and standard errors predicting correspondingly similar or dissimilar sale prices of homes through time.

## Introduction

Quigley and his collaborators derived the hybrid price model as a synthesis of two different models of the determinants of prices of durables such as homes [1–3]. One of two models, the hedonic housing price model, predicts the value of a home by statistically comparing its attributes with those of different individual homes across space and/or through time (e.g., [4–8]). This model assumes analytically that each observed home is different from others and thus has been sold once, even while some are the same homes sold twice or more. These resold homes are specifically analysed in a second model, the repeat sales model, which predicts the change in the value of a resold home from the difference in times and the change in its attributes between its original sale and later resale (e.g., [7, 9–11]).

Quigley’s deduction was that a home’s value would be more accurately predicted by a price model in which changes in resold homes are statistically merged with the attributes and times of sale of once-sold ones. In particular if a home’s value is a function of its attributes, then only the changed attributes of a resold home will need to be observed between its sale and resale. Changed attributes will comprise the systematic components of housing quality as distinct from idiosyncratic components that vary between individual homes ([3], p. 4). He succinctly described the hybrid model’s empirical improvement from this after analyzing a dataset of 843 home sales of which 55% were repeat sales during a 12-year period:

The [hybrid price model’s] parameters … [that utilize the additional information for multiple observations on at least some of the dwellings in the sample] differ from those … of the traditional hedonic model, only in their efficiency of estimation … It is clear in this instance that the confidence interval is tighter when the information obtained from multiple sales is utilized in the estimation process. ([3], p. 10)

### Computational Intensiveness of the hybrid price model

Regardless of this potential empirical improvement in predicting prices, a handful of authors aside from Quigley and his collaborators have calibrated a hybrid price model with their data for houses, artworks or wines [12–17]. Even these authors write about its computational intensiveness that may have discouraged researchers from using it (e.g., [13, 17]). The methodological contribution of this study is the demystification of its computational intensiveness if this is a reason for its underuse in comparison with two other price models. This study demonstrates how to extract information from a repeat sales model and to merge it with that in a hedonic price model for calibrating Quigley’s hybrid price model in Microsoft Excel.

The theoretical contribution of this study is the evaluation of whether a calibrated hybrid model yields improved statistical predictions of changes in house prices in two neighbourhoods during a 30-year period, just in case marginal empirical improvement after all that work is another reason for its underuse. The hypotheses in accordance with Quigley’s conclusion are for a hybrid price model and a corresponding hedonic price model to have statistically-similar typical predictions of house prices, but for the hybrid model to have more precise predictions of those house prices than the hedonic model ([3], p. 10). This is the result in a recent study of 507,218 house sales of which one-half were repeat sales in Perth, Australia, during a seven and one-half-year period, as “the inclusion of repeat-sales information into the longitudinal hedonic estimation model is able to enhance efficiency of estimators” ([14], p. 660). Similarly, it “is the most accurately measured, according to the confidence band around the price index” in another recent study of 22,958 house sales in Mandurah, Australia, between 1994 and 2007 ([15], p. 97). The result is similar in the same author’s study of 14,102 auction sales of Australian wine during 1988 to 2000 [13].

Even so, at least one study cautions about three models’ virtually-identical predictions of house prices in a neighbourhood, for example, “[i]f no mis-specifications are made in the hedonic equation …, the hybrid method is a non-singular transformation of the hedonic measure, making the estimators the same” ([15], p. 96). Moreover, empirical unimprovement may also stem from a weak single sales hedonic model and/or repeat sales model, as three studies applying the hybrid model have different results than the hypothesized ones [12, 16, 17].

### Empirical application of the hybrid price model

One of three studies with different results for a hybrid model than the hypothesized ones infers from graphical evidence that, “[i]t appears to overstate the 18-year house price appreciation in Oakland [CA]” in 27,606 house sales of which 3,342 were repeat sales ([16], p. 63). Second is a study of 1,665 once-sold and 174 repeat-sold Picasso prints between 1988 and 1995. It has a lower 95% confidence interval (CI) for the hybrid model’s predicted prices than those of the corresponding hedonic model, but “differently from what [was] expected, a smaller one than [the] corresponding repeat sales model” ([12], p. 135). The authors do not proceed to resolve these differences between models’ regression coefficients. They do not do this even though three of 15 half-years may have statistical differences between predicted prices at the hedonic model’s lower bound of its 95% CI and the hybrid model’s upper bound of its 95% CI. Another study of 8,218 one-time sales of paintings and 2,207 repeat sales of paintings by the same artist during 2000 to 2016 has regression coefficients for attributes of paintings and times of sale in hybrid and hedonic models. Coefficients in two models are monotonically related but almost all may be statistically significantly different based on tabulated standard errors for both semi-annual and annual analyses [17].

In comparison with these studies, therefore, this study will evaluate the empirical improvement of a hybrid price model after calibrating it with a rich dataset in which one-half of 2,708 house sales are resales of homes for a second time, a third time, and so on up to a tenth time. These are all sales of inhabitable houses through the Multiple Listing Service (MLS) in two inner-city neighbourhoods in Windsor, Ontario, between the beginning of 1986 and end of 2018. A first innovation of this study is the comparison of up to three models’ out-of-sample predictions of observed house prices or changes in prices of 149 houses sold in the neighbourhoods since July 2017, in addition to standard in-sample predictions [18]. These predictions are by calibrated hybrid and single sales models of 2,559 house sales in two neighbourhoods between January 1986 and June 2017. These two models will require a single new time-coefficient for their out-of-sample predictions. A third calibrated model of 1,284 repeat house sales will be used for in-sample predictions, but not out-of-sample ones if recalibration is required for including times of new resold homes’ previous sales [19]. Out-of-sample predictions are more practical with a repeat sales model when both the sale and subsequent resale of a resold home occur during the original study period or after it during a forecast period.

A second innovation exploits the dataset’s coding of selected attributes of the dwelling unit and neighbourhood of each sold home at time of sale or resale. The hybrid model has statistical controls for covariance not only between resold homes’ times of sale and resale as is done in other studies. It also controls for covariance between changes in their attributes of the dwelling unit and neighbourhood at sale- and resale-times. Parenthetically, the studied neighbourhoods are only somewhat relevant herein but note that 540 observed sold houses via the MLS in one neighbourhood, Glengarry, are 61% of up to 882 single-detached or duplex houses in the neighbourhood. A corresponding 760 observed sold houses via the MLS in the other neighbourhood, Wellington-Crawford, are 66% of up to 1,152 single-detached or duplex houses in the neighbourhood.

Subsequent sections in this study will first describe the required organization of observed sold and resold homes’ data. This will be followed by the sequential instantiations of four array formulas including a multiple linear regression as preparations for running a hybrid housing price model as a feasible generalized least squares regression with rescaled data. The last section compares the hybrid model’s predictions of changes in house prices with those by a hedonic housing price model and a repeat sales model in this study and other ones. In preparation for these comparisons, the next section formalizes four equations for predicting values of homes if they are sold once or more than once. These equations also specify the organization of observed data in Excel.

### Software for the analysis: Microsoft Excel

Microsoft Excel 2016/365 is the software for this study’s hybrid modelling under the assumption that a researcher’s data will be organized in that format or can be converted to it. Besides, some researchers may rely on Excel for exploratory analyses of relatively large datasets even if they can run statistical analysis packages. Our dataset is a relatively large one of 70 variables for approximately 2,700 houses in two neighbourhoods, even though it is smaller than recent datasets of repeat house sales. These datasets range upwards from approximately 24,000 repeat sales of single-family houses between January 1998 and June 2010 in Louisville, Kentucky [20]; to approximately 100,000 houses and apartments that sold at least twice between 2001 to 2006 in Sydney, Australia, and an approximate quarter million repeat sales between January 1988 and June 2005 in Perth, Australia [14, 21]; and a half million pairs of single family houses sold between July 1985 and September 2004 in twenty US metropolitan areas, and a similar number of owner-occupied homes sold at least twice between January 1993 and December 2006 in the Netherlands [18, 22].

A usefulness of Excel for exploring large datasets is in programming spreadsheet formulas for live analyses that researchers may inspect and verify from one cell to another on a worksheet and from one worksheet to another. The principles behind these formulas may then be transferred to larger-scale analyses. Above and beyond these spreadsheet formulas, Excel’s analytical power is in instantiated array formulas by simultaneously pressing the Control, Shift and Enter (CSE) buttons on the keyboard. The general procedure with an array formula is to plan for results of the operation by blocking a required area on a worksheet, and then to fill this area with results by Ctrl+Shift+Entering a single array formula that has been pasted within that area. Note however this blocked area’s size on a worksheet may not always forewarn about an unworkable matrix operation. The blocked area for Excel’s linear regression array formula used in this study, for example, is only partly related to its limit of 64 independent variables. This limitation has been considered when hypothesizing the attributes causing price differences, and aggregating times of sale into years etc. In other words, the software is now potentially limiting, for example, the testing of seasonality in house sales as in [23] or changes in prices of attributes through time as in [1].

## Methods

Four equations in this section formalize the single sales hedonic price model and the repeat sales model as predictors of the value of a home or the change in its value as a function of its time of sale or resale, and its attributes or the changes in them such as of its dwelling unit and neighbourhood. Each equation refers to an observed home such an i^th^ once-sold one at time t, and/or a j^th^ resold one when either sold for the first time at time t or resold at a later time τ; and Eqs (1) through (3) are different from Eq (4) as the latter correlates differences in prices with changes in attributes and differences in time. Otherwise, four equations are similar in their calculation of implicit prices for a home’s attributes and times of sale by means of parameters from a linear or nonlinear curve-fitting method such as ordinary least squares in this study. They therefore formalize an assumption about each having the same parameters or rescaled values of them for periods of sale or attributes or changes in attributes. This assumption permits the stacking of Eqs (1) through (4) as the mathematical foundation for the hybrid price model. Their stacking consequently also specifies the order of observations in four submatrices on an Excel worksheet.

### Four house price equations

Theoretically, a single sales hedonic housing price model assumes that an (i = 1,…, I) house is sold once at a (t = 1,..,T) time *t*; and its price, $p_{i}^{t}$, is a function of its (k = 1,…,K) attributes at that time, {$x_{k,i}^{t}$}, and the time of sale itself (e.g., year), $d_{i}^{t}$, equalling 1 for the time of sale and zero otherwise [15, 24]:

$$\mathrm{LN}\;p_{i}^{t}=\alpha +\overset{´}{\beta }x_{i}^{t}+\overset{´}{\gamma }d_{i}^{t}+\eta_{i}+e_{i}^{t}$$ (1)

Where ***β*** = {*β*_*k*_} is a K × 1 vector of implicit prices of attributes, and $x_{i}^{t}=\{x_{k,i}^{t}\}$ is a K × 1 vector of observed attributes of an i^th^ house at sale time *t*. ***γ*** is the T × 1 vector of price changes through time and $d_{i}^{t}$ is the T × 1 vector of time-dependent dummy variables for this i^th^ house. And an error term $\varepsilon_{i}^{t}$ for this home is decomposed into a time-independent specification error *η*_*i*_ and a white noise process $e_{i}^{t}$. Note the operational log-linear function in which the log of the price-difference dependent variable is regressed on untransformed independent variables, as opposed to an alternative log-log function–although neither function has a ruling theoretical recommendation [4, 5]. Coefficients of a log-linear model are interpreted as an approximate percentage change in price for a unit change in an independent variable [25, 26].

A single sales hedonic price model does not differentiate between once-sold and resold homes when it analyzes resold homes as if they have the uncorrelated data of different once-sold ones. However, if each (j = 1,…,J) home sold more than once is distinguished from the I once-sold homes by its *t* time of original resale and *τ* later time of resale, its price when sold for the first time, $p_{j}^{t}$, is a function of its attributes and sale time at time *t*:

$$LNp_{j}^{t}=\alpha +\overset{´}{\beta }x_{j}^{t}+\overset{´}{\gamma }d_{j}^{t}+\eta_{j}+e_{j}^{t}$$ (2)

and its resold price, $p_{j}^{\tau }$, is a function of its potentially-changed attributes and resale time at time *τ*:

$$LNp_{j}^{\tau }=\alpha +\overset{´}{\beta }x_{j}^{\tau }+\overset{´}{\gamma }d_{j}^{\tau }+\eta_{j}+e_{j}^{\tau }$$ (3)

A repeat sales model contrasts with a single sales hedonic price model by explicitly analyzing the same houses sold at least twice during the study period. It assumes by differencing Eq (3) from Eq (2) that a j^th^ home’s difference in resale price at time *τ* versus sale price at time *t*, ($p_{j}^{\tau }-p_{j}^{t}$), is a function of its change in each k^th^ attribute between these times, ($x_{k,j}^{\tau }‐x_{k,j}^{t}$), and the time between sale and resale, ($d_{j}^{\tau }‐d_{j}^{t}$), coded (-1) for time of sale, 1 for time of resale, and zero otherwise in a time-dependent dummy variable, $\delta_{j}^{\tau }$:

$$\left(\mathrm{LN}p_{j}^{\tau }-\mathrm{LN}p_{j}^{t}\right)=\overset{´}{\beta }\left(x_{j}^{\tau }-x_{j}^{t}\right)+\overset{´}{\gamma }\delta_{j}^{\tau }+e_{j}^{\tau }-e_{j}^{t}$$ (4)

Where ***β*** = {*β*_*k*_} is now a K × 1 vector of implicit prices of changes in attributes, and $x_{j}^{t}=\{x_{k,j}^{t}\}$ and $x_{j}^{\tau }=\{x_{k,j}^{\tau }\}$ are K × 1 vectors of observed attributes of a j^th^ house at sale and resale times. ***γ*** is still the T × 1 vector of price changes through time but $\delta_{j}^{\tau }$ is the T × 1 vector of time-differenced dummy variables for a j^th^ house. And the error terms for a j^th^ house are white noise processes of $e_{j}^{t}$ at time t and $e_{j}^{\tau }$ at time *τ*.

### Observations’ data

**Fig 1:**
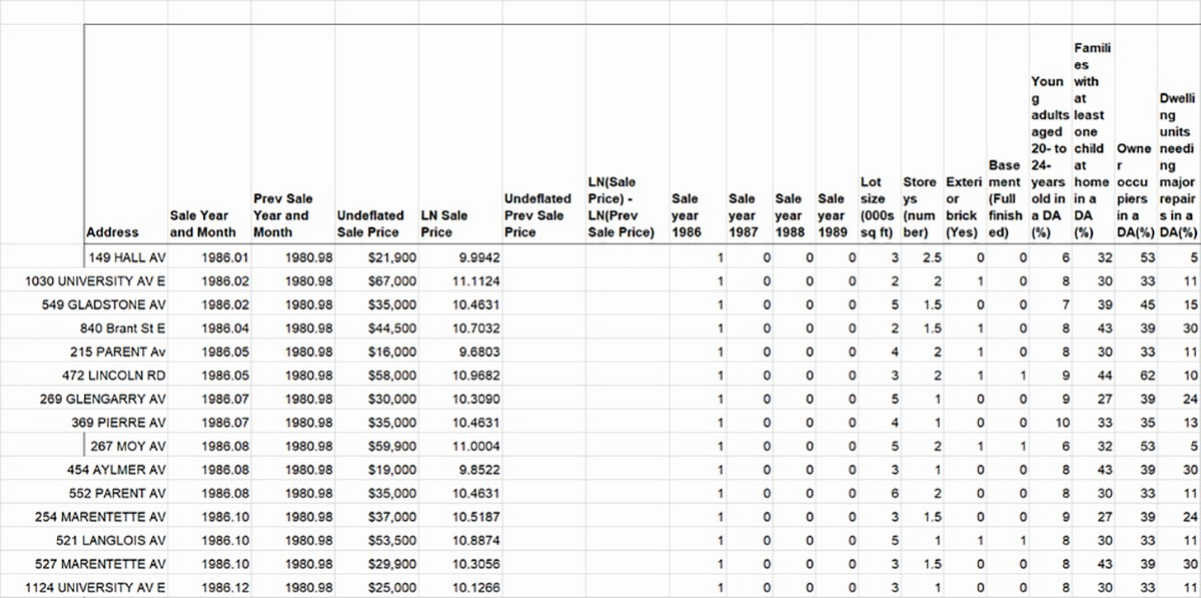
Selected once-sold homes and variables in the dataset.

If an initial focus is on the practical implications of these four equations for organizing observations’ data (where a subsequent focus is on their statistical properties for derivation of the hybrid price model), this study has data for I = 538 once-sold homes in a first set of rows on a worksheet. A second set of rows has the subset of J = 737 resold homes that are sold for the first time, and which are included with the I once-sold homes. A third set of rows has J = 1,284 resold homes when they are sold for the second time, the third time, and so on. A fourth set of rows contains the differences between the sale prices and attributes of J = 1,284 resold homes each time they are resold. Note in Fig 1 the dummy codings of the previous sale year and month as 1980.98 for a once-sold home and (not shown) 1980.99 for the first sale of a resold one. These dummy codings or the observed ones for sale year and month are used for ordering sold houses. The addresses of resold homes are identified in the dataset by means of Excel’s pivot table procedure, its INDEX and MATCH functions with helper columns, or a manual sort of addresses. Then by also using INDEX and MATCH functions, each same home’s change in an attribute or difference in a sale price is calculated between values of a variable in each sale year and month, and previous sale year and month.

In general, therefore, sold homes are in the rows of the worksheet, and their variables including date(s) of sale, undeflated sale price(s), time of sale (and previous sale), and attributes of the dwelling unit and neighbourhood (or changes in them) are in the columns (Fig 1). Note that once-sold homes in the figure have neither previous sale prices nor changes in sale prices, and thus they have blank columns for these variables. Year, month and day of sale are aggregated to the calendar year. The binary coding of I once-sold homes’ times of sale is 1 in the year of sale, and zero before and after. The J homes have the same coding of this variable for each applicable time of sale. As also already mentioned, the time variable for J homes with a calculated difference (in natural logs) between resale price and previous sale price at each time of sale, is coded as (-1) in its year of previous sale, 1 in its year of resale, and zero in remaining years. The J homes sold and resold during the same calendar year have the next coded year as the year of resale to minimize the effect of a brief holding period on the analysis [22].

In addition, eight attributes of the dwelling unit and eight attributes of the neighbourhood are analysed in this study because of their independence from other attributes, changeableness and variability between houses, and potential measurable effect on change in sale price in an inner-city neighbourhood. These attributes are coded as atemporal binary or scale variables, and so each one is assumed to have enduring implicit prices throughout the study period. They are also not disaggregated into several dummy variables for hypothesizing an overall non-linear correlation with the home’s value, such as might be done for number of storeys or condition of a basement. Rather, each attribute or change in an attribute has an additional dummy variable with the value of that attribute or change in it for a sold or resold home in the Wellington-Crawford neighbourhood and zero for one in Glengarry. A last dummy variable has unities for I once-sold homes and J resold ones, and zeroes for J resold homes’ differences; where the data in this column may calculate, respectively, a constant intercept in the regression of the former, and none in the regression of the latter.

### Running the multiple regressions

**Fig 2:**
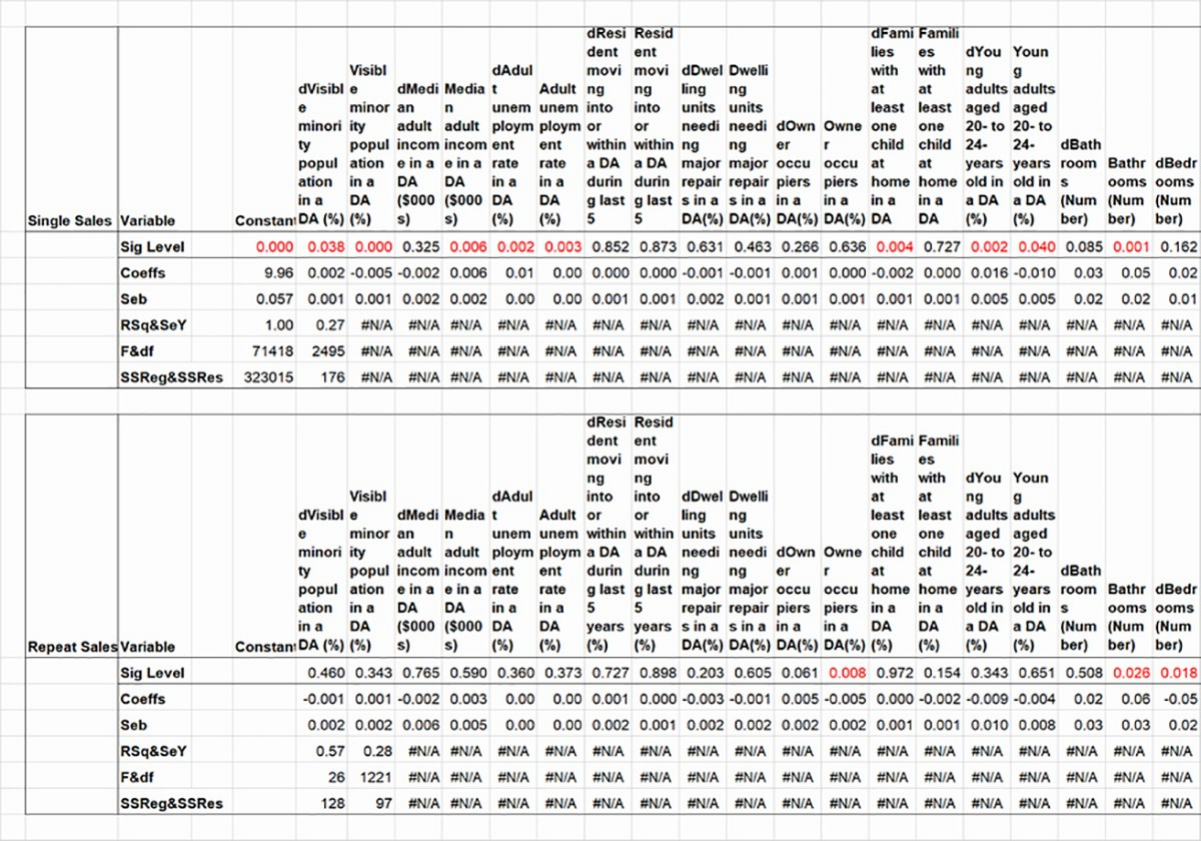
Two models’ selected linear regression results.

Least squares regression coefficients for two submatrices of data in this study are calculated with Excel’s LINEST array formula (Fig 2). One submatrix includes the data for stacked Eqs (1) through (3), and its partial output is labelled as Single Sales in the figure; and the second includes that for Eq (4), labelled as Repeat Sales in the figure. Operationally, LINEST requires a blocked area on a worksheet for its results that has five rows, and columns equal to the (K + T) number of independent variables plus one for the constant intercept if it is calculated. The constant intercept of the single sales regression may be calculated by either requesting it in the LINEST array formula, or not requesting it and instead utilizing the aforementioned additional column of unities as in the result in the figure. The repeat sales regression has no constant intercept, and so it is not requested in the LINEST array formula in the result in the figure.

Note in the resulting output shown in the figure, first, the reversed order of the regression coefficients and their standard errors in the blocked area’s first two rows, in comparison with the order of the analysed variables on the worksheet in Fig 1. Column headings for variables are therefore reversed to label the displayed results. More importantly as explained below, these reversed orders will be computer-programmed into calculations of residuals from the regression with these variables’ values and coefficients. Note second that the regression’s summary statistics including the R-squared, F statistic, and degrees of freedom are displayed in the blocked area’s first two columns of the third through fifth rows (although these should be ignored for both models, as the method explained above for calculating a constant intercept has inflated LINEST’s displayed values of R-squared and the F statistic).

### Calculating residuals from the regressions

Sale prices of sold and resold homes or the differences between them have now been regressed on their attributes or the changes in them and their times of sale in accordance with Eqs (1) through (3), and Eq (4). If the focus is now on the statistical properties of these stacked equations for the derivation of the hybrid price model, the ensuing analysis will update the error structure of the single sales results from stacked Eqs (1) through (3) by removing unobserved specification errors due to dependencies between the repeat sales of the same homes calculated in Eq (4). The derivation by [24] of these error terms for inclusion in a covariance matrix corresponds with the analysis of the random effects model by [27]. The assumptions for an i^th^ or j^th^ home, h = {i, j}, are that its time-dependent residuals, {$e_{h}^{t}$}, from linear regression have a variance, $\mathrm{Var}(e_{h}^{t})=\sigma_{e}^{2}$, while their mean is zero, $E(e_{h}^{t})$ = 0, and their covariance with other homes’ attributes is also zero, $\mathrm{Cov}(e_{i}^{t},e_{j}^{t})$ = $\mathrm{Cov}(e_{h}^{t},e_{h}^{\tau })$ = 0. Similar assumptions apply to its time-independent residuals, {*η*_*h*_}: Var(*η*_*h*_) = $\sigma_{\eta }^{2}$, while E(*η*_*h*_) = 0 and $\mathrm{Cov}(e_{h}^{t},\;\eta_{j})$ = 0. Then the (I + 2J) × (I + 2J) covariance matrix of the error structure is:

$$\Omega =\left[\begin{array}{ccc} (\sigma_{\eta }^{2}+\sigma_{e}^{2})=\sigma_{\varepsilon }^{2}I & 0 & 0\\ 0 & (\sigma_{\eta }^{2}+\sigma_{e}^{2})=\sigma_{\varepsilon }^{2}I & (-\sigma_{e}^{2})I\\ 0 & (-\sigma_{e}^{2})I & 2\sigma_{e}^{2}I \end{array}\right]$$ (5)

If $\sigma_{\varepsilon }^{2}$ is the variance of the error of the single sales regression of stacked Eqs (1), (2) and (3), where $\sigma_{\varepsilon }^{2}=(\sigma_{\eta }^{2}+\sigma_{e}^{2})$, it may be estimated with the residuals, {${\hat{\varepsilon }}_{n}$}, from the ordinary least squares regression for all (n = 1,…,N (= I + J)) once-sold and resold homes. Note therefore as clarified below that the first row or column of the covariance matrix refers to statistics of once-sold homes and resold homes sold for the first time, whereas the second row or column refers to those of resold homes sold for the second time and so on. The summary statistic also has an adjustment for lost degrees of freedom for K coefficients of homes’ attributes, T coefficients of their aggregated times of sale, and the constant intercept:

$${\hat{\sigma }}_{\varepsilon }^{2}=\left(\frac{1}{N-K-T-1}\right)\sum_{n=1}^{N}{\hat{\varepsilon }}_{n}^{2}$$ (6)

Correspondingly, $\sigma_{e}^{2}$ is one-half of the variance of the error of the repeat sales regression of Eq (4), and so it may be estimated with the (j = 1,…,J) residuals, {${\hat{\xi }}_{j}$}, from this regression, also with an adjustment for lost degrees of freedom for K coefficients of homes’ attributes, and T coefficients of their aggregated times of sale, but no constant intercept:

$${\hat{\sigma }}_{e}^{2}=\frac{1}{2}\left(\frac{1}{1-K-T}\right)\sum_{j=1}^{J}{\hat{\xi }}_{j}^{2}$$ (7)

A repeat sales model with a relatively lower error variance has more accurately estimated its dependent variable, and thus it has more information to contribute about this to a hybrid model as a recalibration of a single sales hedonic model in the next section.

Operationally, the reversed order of the columns of input data and columns of output results on an Excel worksheet complicates the potentially simple calculation of residuals from the single sales regression and the repeat sales regression. If there was no need to reorder one set of columns, a SUMPRODUCT formula with appropriate ranges of relative cell references for each home’s data and absolute cell references for the regression coefficients would multiply variables’ values by their coefficients and sum these products for a predicted value. Thus required is either a virtual sort or manual sort of cells across one set of columns to have the same order as those in another set of columns. A manual sort may however be counter-productive unless the LINEST procedure will not be rerun, as an efficiency of this procedure is the usability of its latest output in cells by subsequent procedures.

A virtual sort is instead implemented with OFFSET by programming a dragged and dropped formula from left to right in a row of cells across a range of columns so that it identifies the cell with the same position from right to left in the range of columns in the same row. The values of these virtually-sorted cells, such as containing the regression coefficients, are now the ones multiplied by the values of each house’s variables and summed in a SUMPRODUCT procedure that can be dragged and dropped down a suitable column on the worksheet. If desired in the same column, each home’s predicted LN sale price or change in LN sale price may be subtracted from its respective observed value, and then this difference, squared. After summing these squared residuals, the adjustments for the degrees of freedom (in a cell in LINEST’s output) are made for calculating estimates of the error variance in Eqs (6) and (7). Incidentally, each error variance may be transformed into a root mean squared error, which may then be transformed into a simple R-squared of the correlation between observed values and predicted values from a regression model.

### Analyzing the covariance matrix

**Fig 3:**
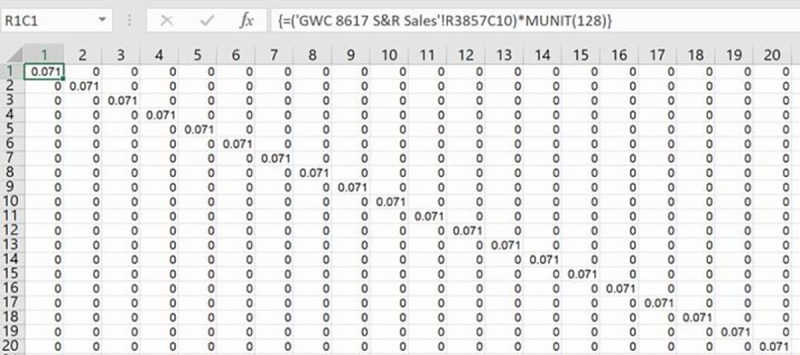
Partial covariance matrix.

These estimates of the error variance are elements for creating the estimated covariance matrix after multiplying them by identity matrices across cells in specific rows and columns and filling remaining cells with zeroes. As this covariance matrix only has active values in its diagonal cells and predictable off-diagonal cells, and zeroes elsewhere, a proportionally-smaller version of it is created and subsequently inverted and decomposed [13]. Fig 3 displays, for example, the creation of the identity matrix’s multiplication in the top-left cell of the covariance matrix in Eq (5). It is created with Excel’s MUNIT array formula for one-tenth (128) of the full (1,275) rows for the single sales I homes. Note that rows and columns in the worksheet are displayed in R1C1 reference style for easier positioning of the cursor using the little window on the left side of the formula bar, such as when blocking an area for MUNIT’s results. Values in remaining cells on the diagonal of the covariance matrix are calculated with the appropriate estimate of the error variance via MUNIT for the next 128 rows and columns, and so on for the next 128 rows and columns after those. Active off-diagonal values in the middle-right and bottom-middle cells of the covariance matrix are similarly calculated with the appropriate error variance programmed in the MUNIT array formula.

**Fig 4:**
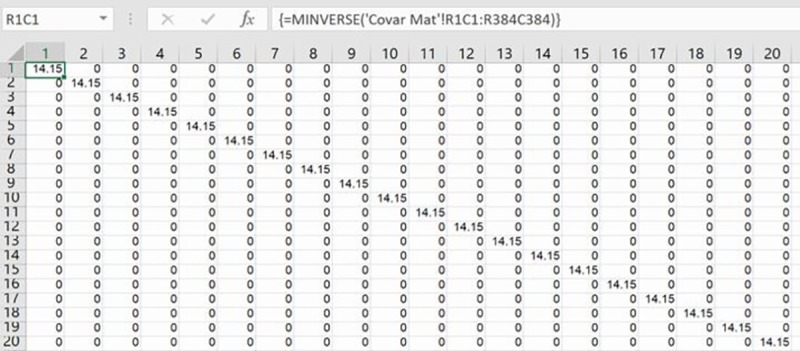
Partial inverted covariance matrix.

**Fig 5:**
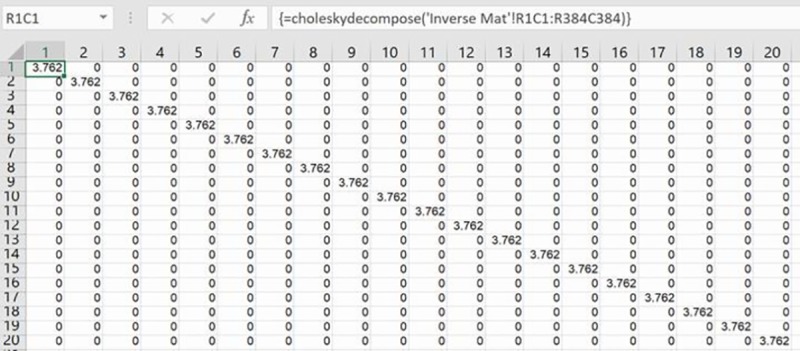
Partial Cholesky decomposition P-values.

**Fig 6:**
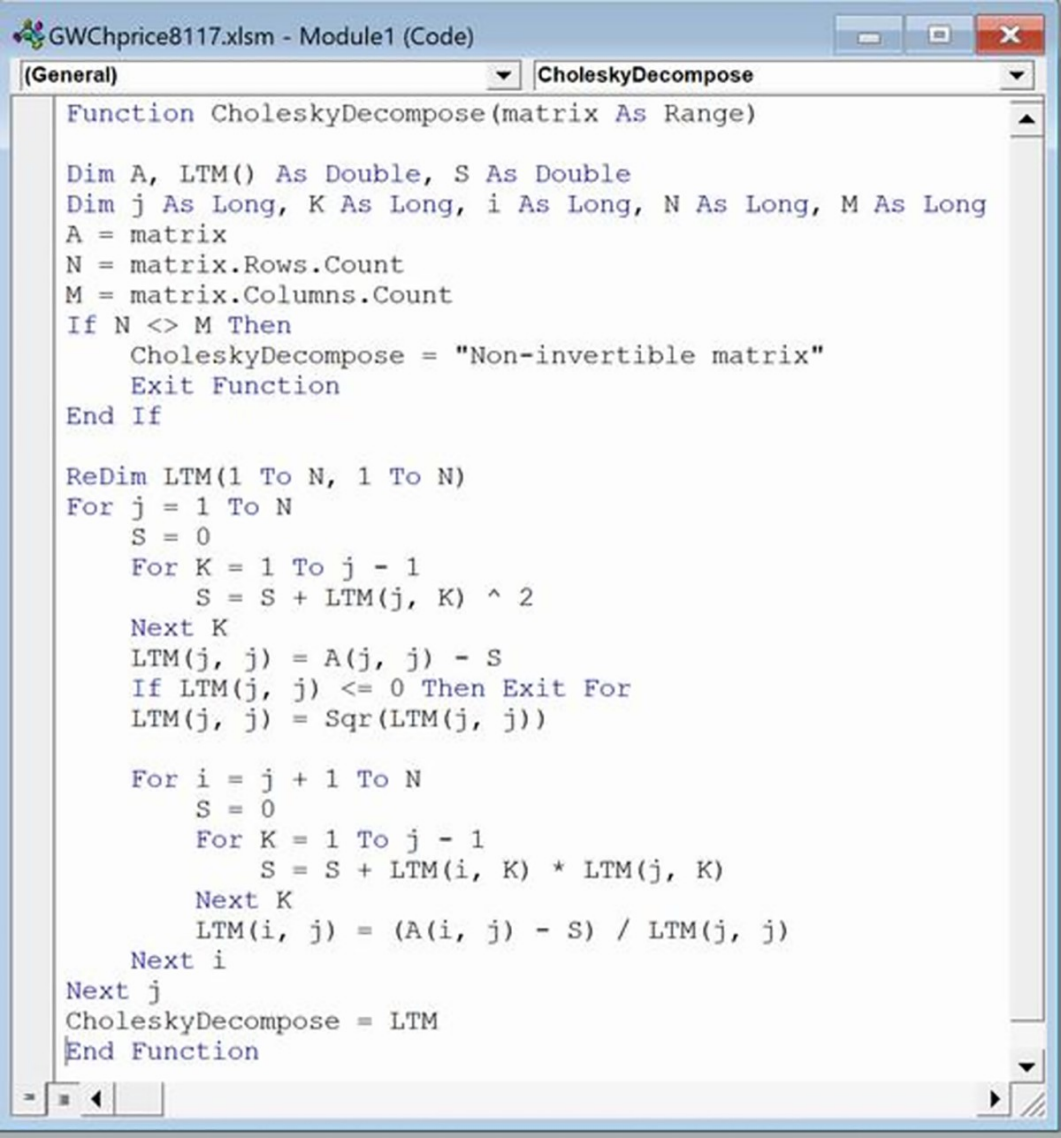
Cholesky decomposition in Excel VBA.

The inversion of the estimated covariance matrix, $\hat{\Omega }$, and the Cholesky decomposition of this inverted matrix, ${\hat{\Omega }}^{-1}$, for calculating its P-value matrix such that $\overset{´}{PP}={\hat{\Omega }}^{-1}$, are the final preparatory steps in the hybrid modelling procedure. Excel’s MINVERSE array formula inverts the covariance matrix after blocking the required area for its results on a new worksheet (Fig 4). The results of a Cholesky decomposition are also on a new worksheet after blocking the required area for the results (Fig 5). Cholesky decomposition is not a built-in function in Excel, and so a Visual Basic (VBA) program of it has been loaded from the internet into a local copy of Excel via the Developer tab (Fig 6). Instantiation of this array formula with its correct VBA name is then the same as a built-in array formula. Just as rows and columns of the covariance matrix were a proportion of the (I + 2J) single sales and repeat sales houses, so too are those of the P-value matrix from the Cholesky decomposition. These proportional dimensions of the P-matrix preclude its MMULT matrix multiplication with all observations’ data for rescaling them prior to running the hybrid model. However, active values in the P-matrix have predictable on-diagonal and off-diagonal locations as summarized in Eq 8; where the first row or column of this matrix in this study represents 1,275 once-sold homes and first-time resold homes, the second row or column represents 1,284 resold homes sold twice or more, and the third row or column represents 1,284 resold homes’ differences:

$$P=\left[\begin{array}{ccc} (3.76)I & 0 & 0\\ 0 & (4.44)I & (2.22)I\\ 0 & (2.22)I & (3.55)I \end{array}\right]$$ (8)

These predictable locations of active cells in the P-matrix enable an elegant solution in Excel for rescaling data of the observed I once-sold homes and J resold homes as if they were multiplied by a complete P-matrix. That is, data in the I (or J) houses’ rows of cells in a column (such as that for LN Sale Price in Fig 1) are, first, array-multiplied by their appropriate P-value; and second, this product is added to the corresponding array-multiplication of data in their J (or I) rows of cells in the same column, with one exception. The exception is that the dependent variable of single sales and resold houses is in the LN Sale Price column in Fig 1, whereas that of the repeat sales is in the (LN Sale Price–LN Previous Sale Price) column; and so their array-multiplications refer to different columns as well as different rows of cells.

Note that either this solution or a MMULT matrix multiplication necessitates a regeneration of the full dataset in columns such as to the right of the existing ones. Hence, if the first new column to the right of the existing ones contains the array formulas for the rescaled dependent variable, it may then be dragged and dropped with amended columns’ references across the remaining new columns. Appropriate P-values are thereby array-multiplied by each value of the independent variables and the already-inserted column of unities or zeroes for calculating a correctly-scaled constant intercept. The hybrid price model is finally calibrated by rerunning the LINEST procedure without a constant on the new rescaled data for Eq (1)’s I once-sold homes, Eq (2)’s J resold homes sold for the first time, and Eq (4)’s J repeat sales. In other words, this final run of LINEST will omit Eq (3)’s J resold homes sold for the second time, the third time and so on. Partial deletions of rows may therefore be required on the worksheet for preparing the required contiguous input data for rerunning the array formula, such as if the P-matrix rescaled data are in the same rows to the right of the original data.

## Results

**Fig 7:**
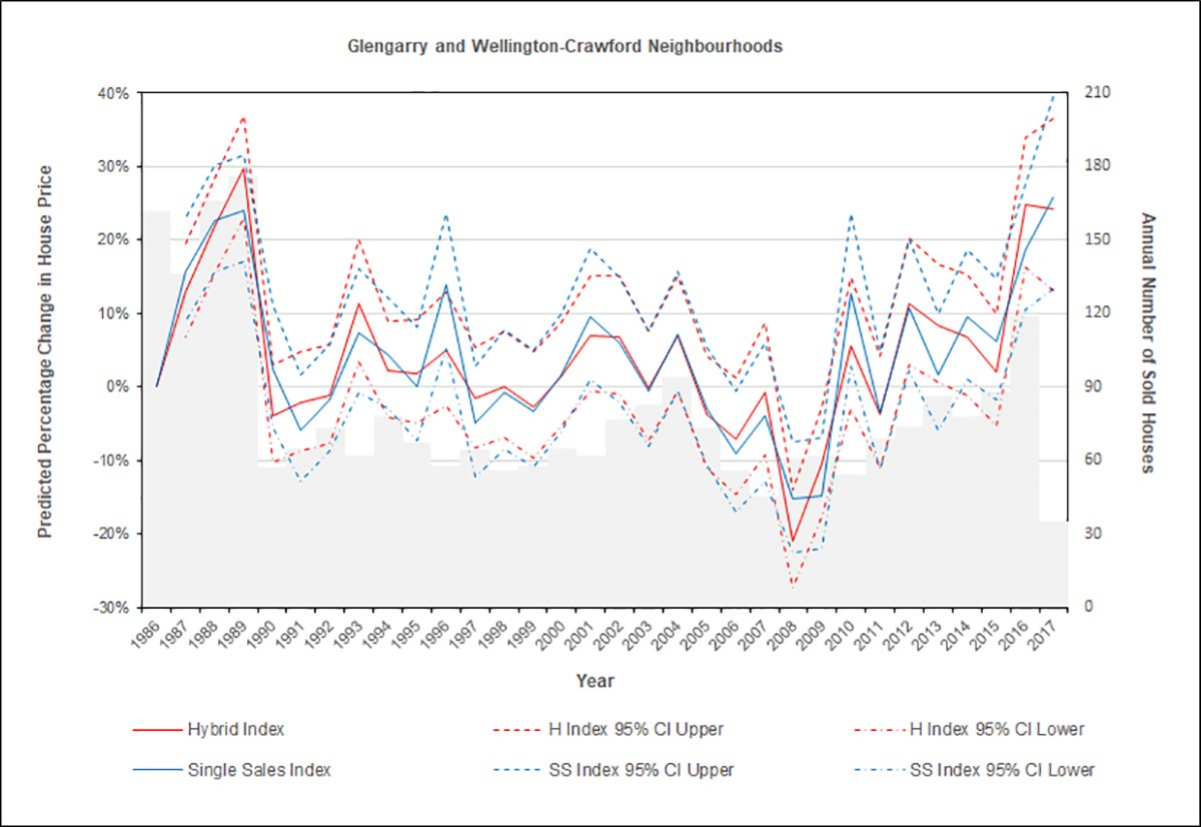
Predicted changes in house prices through time.

The results of the rerun LINEST array formula for the hybrid price model resemble those in Fig 2, and together with 95% confidence intervals of attributes’ values, they are displayed in Table 1 for comparison with those of the single sales hedonic model. Each model’s regression coefficients and standard errors are also used in Fig 7 for calculating 95% confidence intervals of predicted annual percentage changes in prices for 2,559 house sales in two neighbourhoods between 1986 and mid-2017. In a comparison of models’ results, the hybrid price model’s statistical results have improved as hypothesized with more precise estimates of prices than those of the single sales hedonic model but statistically the same regression coefficients (Table 1). Each of the hybrid model’s standard errors for 31 statistically-significant regression coefficients of times of sale and resale is lower than the corresponding one of the single sales hedonic model, even though these standard errors have similar values (and each is lower than the corresponding repeat sales model’s one in results available from the authors).

**Table 1:** Two models’ regression coefficients.

Variables				Single Sales Hedonic Model				Hybrid Price Model			
		Attribute 95% CI		Coeff-icient	Coeff Stand-ard Error	Signif-icance Level		Coeff-icient	Coeff Stand-ard Error	Signif-icance Level	
		Lower	Upper								
*Dependent*	Sale Price	$81,257	$83,972								
	Resale Price	$86,243	$89,945								
*Dwelling Unit*	Lot size in 000s of sq. ft.	3.9	4.1	0.049	0.007	0.00	^‡^	0.040	0.009	0.00	^‡^
	WC Dummied lot size in 000s of sq. ft.	2.4	2.5	-0.012	0.008	0.17		-0.003	0.011	0.75	
	Number of bathrooms	1.6	1.7	0.053	0.015	0.00	^‡^	0.065	0.017	0.00	^‡^
	WC Dummied number of bathrooms	0.9	1.0	0.034	0.020	0.08		0.026	0.022	0.23	
	Number of bedrooms	3.5	3.6	0.037	0.010	0.00	^‡^	0.051	0.011	0.00	^‡^
	WC Dummied number of bedrooms	2.0	2.2	0.017	0.012	0.16		-0.005	0.014	0.73	
	Number of garages	0.4	0.5	0.057	0.012	0.00	^‡^	0.067	0.014	0.00	^‡^
	WC Dummied number of garages	0.2	0.3	0.008	0.016	0.62		0.005	0.019	0.81	
	Number of storeys	1.8	1.8	0.172	0.019	0.00	^‡^	0.164	0.024	0.00	^‡^
	WC Dummied number of storeys	1.0	1.1	-0.120	0.025	0.00	^‡^	-0.142	0.030	0.00	^‡^
	Central air conditioning, 1 = yes	0.4	0.4	0.111	0.018	0.00	^‡^	0.123	0.019	0.00	^‡^
	WC Dummied central air conditioning, 1 = yes	0.2	0.3	0.060	0.023	0.01	*	0.045	0.025	0.07	
	Exterior brick finish, 1 = yes	0.4	0.4	0.175	0.020	0.00	^‡^	0.141	0.025	0.00	^‡^
	WC Dummied exterior brick finish, 1 = yes	0.2	0.2	-0.049	0.025	0.05	*	-0.011	0.031	0.73	
	Full finished basement, 1 = yes	0.3	0.3	0.059	0.022	0.01	^†^	0.017	0.021	0.41	
	WC Dummied full finished basement, 1 = yes	0.2	0.2	0.011	0.027	0.70		0.045	0.025	0.08	
*Neighbour-hood*	Median annual income of adults in a DA in $000s	$21	$21	0.006	0.002	0.01	^†^	0.008	0.002	0.00	^†^
	WC Dummied median annual income of adults in a DA in $000s	$12	$13	-0.002	0.002	0.33		-0.005	0.003	0.05	*
	Percentage of dwelling units in a DA needing major repairs	11%	12%	-0.001	0.001	0.46		-0.001	0.001	0.23	
	WC Dummied percentage of dwelling units in a DA needing major repairs	6%	6%	-0.001	0.002	0.63		-0.002	0.002	0.27	
	Percentage of households in a DA with at least one child at home	48%	49%	0.000	0.001	0.73		-0.001	0.001	0.44	
	WC Dummied percentage of households in a DA with at least one child at home	27%	29%	-0.002	0.001	0.00	^†^	-0.002	0.001	0.02	*
	Percentage of owner occupiers in a DA	35%	37%	0.000	0.001	0.64		-0.002	0.001	0.03	*
	WC Dummied percentage of owner occupiers in a DA	20%	21%	0.001	0.001	0.27		0.003	0.001	0.01	*
	Percentage of residents moving into or within a DA during 5-year period	57%	58%	0.000	0.001	0.87		-0.001	0.001	0.24	
	WC Dummied percentage of residents moving into or within a DA during 5-year period	33%	35%	0.000	0.001	0.85		0.001	0.001	0.16	
	Percentage of visible minority population in a DA	30%	31%	-0.005	0.001	0.00	^‡^	-0.003	0.001	0.00	^†^
	WC Dummied percentage of visible minority population in a DA	17%	18%	0.002	0.001	0.04	*	0.002	0.001	0.04	*
	Percentage of young adults aged 20- to 24-years old in a DA	10%	10%	-0.010	0.005	0.04	*	-0.015	0.005	0.00	^†^
	WC Dummied percentage of young adults aged 20- to 24-years old in a DA	7%	7%	0.016	0.005	0.00	^†^	0.017	0.006	0.00	*
	Percentage unemployment rate in a DA	8%	9%	-0.005	0.002	0.00	^†^	-0.004	0.002	0.01	*
	WC Dummied percentage unemployment rate in a DA	5%	5%	0.006	0.002	0.00	^†^	0.004	0.002	0.01	*
*Year of Sale*	2017			1.157	0.053	0.00	^‡^	1.138	0.048	0.00	^‡^
	2016			0.928	0.036	0.00	^‡^	0.921	0.036	0.00	^‡^
	2015			0.756	0.040	0.00	^‡^	0.700	0.038	0.00	^‡^
	2014			0.697	0.041	0.00	^‡^	0.680	0.039	0.00	^‡^
	2013			0.606	0.040	0.00	^‡^	0.614	0.038	0.00	^‡^
	2012			0.590	0.041	0.00	^‡^	0.533	0.039	0.00	^‡^
	2011			0.487	0.043	0.00	^‡^	0.427	0.040	0.00	^‡^
	2010			0.524	0.047	0.00	^‡^	0.464	0.043	0.00	^‡^
	2009			0.405	0.045	0.00	^‡^	0.410	0.043	0.00	^‡^
	2008			0.565	0.045	0.00	^‡^	0.521	0.042	0.00	^‡^
	2007			0.731	0.050	0.00	^‡^	0.755	0.046	0.00	^‡^
	2006			0.770	0.046	0.00	^‡^	0.762	0.043	0.00	^‡^
	2005			0.865	0.042	0.00	^‡^	0.835	0.040	0.00	^‡^
	2004			0.896	0.039	0.00	^‡^	0.873	0.037	0.00	^‡^
	2003			0.826	0.040	0.00	^‡^	0.806	0.038	0.00	^‡^
	2002			0.833	0.041	0.00	^‡^	0.807	0.038	0.00	^‡^
	2001			0.774	0.042	0.00	^‡^	0.741	0.037	0.00	^‡^
	2000			0.682	0.040	0.00	^‡^	0.674	0.035	0.00	^‡^
	1999			0.666	0.041	0.00	^‡^	0.660	0.037	0.00	^‡^
	1998			0.700	0.042	0.00	^‡^	0.687	0.037	0.00	^‡^
	1997			0.707	0.040	0.00	^‡^	0.687	0.036	0.00	^‡^
	1996			0.758	0.041	0.00	^‡^	0.703	0.037	0.00	^‡^
	1995			0.627	0.039	0.00	^‡^	0.655	0.035	0.00	^‡^
	1994			0.626	0.037	0.00	^‡^	0.636	0.033	0.00	^‡^
	1993			0.584	0.040	0.00	^‡^	0.614	0.038	0.00	^‡^
	1992			0.513	0.038	0.00	^‡^	0.506	0.034	0.00	^‡^
	1991			0.530	0.039	0.00	^‡^	0.518	0.035	0.00	^‡^
	1990			0.591	0.041	0.00	^‡^	0.540	0.035	0.00	^‡^
	1989			0.567	0.029	0.00	^‡^	0.579	0.027	0.00	^‡^
	1988			0.351	0.030	0.00	^‡^	0.318	0.027	0.00	^‡^
	1987			0.146	0.031	0.00	^‡^	0.122	0.029	0.00	^‡^
	1986			0.000				0.000			
*Constant*				9.957	0.057	0.00	^‡^	10.053	0.068	0.00	^‡^
	Mean Standard Error of Coefficients			0.041				0.038			
	R-Squared			65%				96%			
	Degrees of Freedom			2495				2495			
	Number of Houses			2559				2559			
			Significance Level less than 0.001				^‡^				
			Significance Level less than 0.01				^†^				
			Significance Level less than 0.05				*				

Moreover, refined values of the hybrid model’s regression coefficients and their standard errors culminate in a significant empirical improvement in its explained variation of observed homes’ prices over two other models [28]. Its simple squared correlation of 96% between observed sale prices and predicted prices by regression coefficients is 31% higher than the single sales hedonic model and 45% higher than the repeat sales model. It is also higher than 88% in another study, even though our single sales hedonic model’s 65% and repeat sales model’s 51% are lower than 83% and approximately 90%, respectively, for those models in that study ([1], p. 55). They similarly differ from the respective ones of 90%, 73%, and 74% in another study ([3], p. 8).

In addition, the hybrid model has a higher simple R-squared of 94% than the single sales model’s 55% in out-of-sample tests that correlate predicted and observed prices or LN differences in prices of 25 once-sold houses and 124 resold houses since July 2017 to December 2018. Note comparable predictions by the repeat sales model would require its recalibration for all resold homes except two with a sale and subsequent resale occurring since July 2017. More simply, regression coefficients of the hybrid and single sales models for the 1986 to 2017 sale-years in Table 1 are each augmented in these out-of-sample predictions with a linearly-filled coefficient for 2018 from five previous years’ coefficients in Excel. The hybrid model therefore performs better with this extrapolation in its predictions of recent sale prices (between undeflated $167,381 and $186,163 in a 95% confidence interval) of homes that have similar attributes of the dwelling unit and neighbourhood as in earlier years, except for their higher likelihoods of having central air conditioning and being located in a neighbourhood with families having at least child at home.

Even so, differences between two models’ in-sample predicted house prices through time are frequently not noticeable in Fig 7 between the 95% confidence intervals calculated with a model’s regression coefficients and standard errors; and this is in contrast to those already quoted from another study ([3], p. 10). Average standard errors for sale-years’ regression coefficients of 0.038 for the hybrid model, 0.041 for the single sales model, and 0.049 for the repeat sales model are also much closer to each other than the corresponding ones of 0.032, 0.053, and 0.074 in another study ([13], p. 6). Hence, the hybrid model has marginally-more statistically-confident predictions of three subtrends than those of two other models. Three subtrends are summarized as, first, average predicted annual percentage changes in house prices increasing with increasing numbers of sold homes (in the grey background of the figure) during the late-1980s; second, a 20-year period of fluctuating small increases and possibly ‘larger’ decreases in prices until 2011; and a third period since then with increases in prices again. Note these statistics are calculated with an average of 77 house sales in a calendar year, and a minimum of 35 annual sales up to a maximum of 176 annual sales. Low numbers of observed house sales may thus coincidentally preclude a seasonal analysis, such as of quarterly periods of sales even if the additional required time-dependent dummy variables did not exceed the executable limits of Excel.

Otherwise, this study’s innovative rescaling of values for attributes of the dwelling unit and neighbourhood has not contributed as much to the hybrid model’s empirical improvement as the standard rescaling of values for times of sale and resale. This however may be due to the repeat sales model’s less new statistical information about correlations of changes in attributes with changes in prices. It has nine statistically-significant coefficients at less than the 5% level for 32 attributes of the dwelling unit and neighbourhood including their dummy variables. Seven of nine from the repeat sales model are also among the hybrid model’s 19 statistically-significant coefficients for the attributes at less than the 5% level; and these 19 also include 16 of 19 from the single sales model (with six same as repeat sales model), plus two independent variables that are not statistically significant in either model. Last, the hybrid model and single sales model have similar 0.009 and 0.011 for their respective average standard errors of 19 significant coefficients for the attributes’ variables; and only one of the former’s standard errors is lower than that of the latter.

More substantively, the hybrid model’s statistically-significant regression coefficients for sold homes’ attributes of the dwelling unit and neighbourhood affirm standard-hypothesized relationships between homes’ (re)sale house prices and especially attributes of the dwelling unit’s livable area. For example, an attribute with a sizeable statistically-significant coefficient and dummy coefficient in the hybrid price model is for an additional storey predicted to add 21% to the resale price of a home in Glengarry and 13% in Wellington-Crawford. In addition, statistically-significant coefficients in the hybrid price model for three attributes of the neighbourhood differentiate between lower-income university-student residents of one neighbourhood and lower-income unemployed renter residents of the other. For example, one percent increase in young adults aged 20- to 24-years-old in a DA is predicted to have 1.4% decrease in a home’s (re)sale price in Glengarry, but 0.1% increase in Wellington-Crawford where young adults are more likely to be students than in the former neighbourhood. Relatedly, one percent increase in adult unemployment rate or in owner-occupier residents in a DA will have a respective 0.4% or 0.2% decrease in a home’s (re)sale price in Glengarry, but a same 0.1% increase in Wellington-Crawford where the unemployed and renters are more likely to be students. Either way, additional lower-income residents in neighbourhoods are associated with lower prices of homes, as a decrease of one thousand dollars in median adult income in a DA is predicted to have 0.8% decrease in a home’s (re)sale price in Glengarry and 0.2% decrease in Wellington-Crawford.

## Discussion

This study’s demonstration of the calibration of Quigley’s hybrid price model in Excel with transferable procedures into other statistical software has simplified its computation if the intensiveness of this is a reason for its underuse. The hybrid model was operationalized in this study by statistically rescaling data for the prices, times of (re)sale and attributes of the dwelling unit and neighbourhood of 1,275 once-sold ones with those of 1,284 resold homes during a 30-year period. In addition to this long study period, an innovation was in rescaling the data for the attributes of homes as well as their times of sale, although the former subsequently contributed less than the latter to the hybrid price model’s marginally improved predictions of sold house prices in two inner-city neighbourhoods. Data for these observations’ 70 variables were analyzed within the executable limits of a linear regression procedure in Excel, but a statistical analysis package will be utilized if data are available for refinements such as quarterly periods of sales rather than annual ones, or changes in prices of attributes through time rather than assumed enduring ones. So far, the hybrid price model has had the hypothesized empirical improvement in modelling house prices. First, it statistically explains more of the variation in the house price dependent variable than does a single sales hedonic price model or a repeat sales model. Second, it has more accurate predictions than the former model of changes in both in-sample and out-of-sample house prices through time; and it has more accurate in-sample predictions than the latter model. It achieves these improvements with regression coefficients for homes’ times of sale and resale having marginally lower standard errors than those in the single sales hedonic price model or the repeat sales model.

An already-quoted theoretical reason in the literature for a hybrid price model’s marginal empirical improvement is the lack of covariation between resold homes’ attributes and times of sale in a correctly-specified single sales hedonic price model. Alternatively in this study, new information about this covariation may have been contributed by this study’s repeat sales model as resold homes comprised one-half of the dataset for the hybrid price model. Even so, both the repeat sales model and the single sales hedonic model behind this study’s hybrid price model have poorer goodness of fits than in at least three other studies. Sensitivity analyses are therefore planned in future research with Excel for simulating predictions of homes’ prices under different conditions of numbers of observed one-time sales, proportions of repeat sales, and goodness of fits of single sales hedonic price models and repeat sales models. In conclusion, this study has begun identifying statistical constraints on a hybrid price model’s empirical improvement over two other price models if perceived unimprovement is another reason for its underuse.
